# Erwartete Schulprobleme als Folge der Corona-Schulschließungen im Frühjahr 2020 – Empirische Evidenz zur Bedeutung familialer Ressourcen mittels nichtlinearer Modellierung

**DOI:** 10.1007/s11618-023-01149-9

**Published:** 2023-06-12

**Authors:** Jennifer Lorenz, Sina Ike, Lea Maria Dammann, Dominik Becker, Benjamin Säfken, Alexander Silbersdorff

**Affiliations:** 1 Zentrum für Statistik, Georg-August-Universität Göttingen, Humboldtallee 3, 37073 Göttingen, Deutschland; 2 Professur für Statistik, Georg-August-Universität Göttingen, Humboldtallee 3, 37073 Göttingen, Deutschland; 3 Lehrstuhl für Maschinelles Lernen, Georg-August-Universität Göttingen, Goldschmidtstraße 1, 37077 Göttingen, Deutschland; 4 Professur für Data Science und Angewandte Statistik, Technische Universität Clausthal, Adolph-Roemer-Straße 2a, 38678 Clausthal-Zellerfeld, Deutschland; 5 CIDAS Campus-Institut Data Science, Georg-August-Universität Göttingen, Goldschmidtstraße 1, 37077 Göttingen, Deutschland

**Keywords:** Coronapandemie, Homeschooling, Soziale Ungleichheit, Generalisierte Additive Modelle, Semiparametrische Regression, Covid pandemic, Home Schooling, Social inequality, Generalized Additive Models, Semiparametric Regression

## Abstract

Angesichts der Schulschließungen im Zuge der Coronapandemie standen Schulen, Familien und Kinder in Deutschland im Frühjahr 2020 vor der großen Herausforderung, den Unterricht zu Hause fortzuführen. Der vorliegende Beitrag untersucht die Erwartungen der Eltern, dass innerhalb der kommenden sechs Monate Schulprobleme als Folge des Homeschoolings auftreten werden. In explorativer Herangehensweise werden zur Analyse nichtlineare Modelle eingesetzt. Im Zuge dessen werden nichtlineare Modelle vorgestellt und erläutert, um den Mehrwert im Vergleich zu den in der empirischen Bildungsforschung üblicherweise verwendeten Methoden hervorzuheben. Für die Analyse werden die verwendeten Daten des Nationalen Bildungspanels (NEPS) mit zusätzlichen Datenquellen wie dem COVID-19-Dashboard des Robert Koch-Instituts (RKI) kombiniert. Die Ergebnisse zeigen, dass die elterlichen Erwartungen zukünftiger Schulprobleme insbesondere unter jenen Eltern verbreitet waren, deren Kinder eine niedrige Lesekompetenz und eine geringe Sorgfältigkeit als Teilaspekt der schulischen Anstrengungsbereitschaft aufwiesen. Des Weiteren findet sich ein Zusammenhang zwischen einem niedrigeren beruflichen Status (ISEI) der Eltern und einer zunehmenden Erwartung von Schulproblemen. Weiterhin weisen die kurzfristigen und die langfristigen Sorgen der Eltern hinsichtlich Corona eine positive Assoziation auf und machen Schulprobleme in den Augen der Eltern wahrscheinlicher. Das Ziel dieses Beitrags ist es, neben der erstmaligen Anwendung und Erläuterung von nichtlinearen Modellen in der empirischen Bildungsforschung, die Erwartung hinsichtlich Problemen des Homeschoolings im ersten Lockdown aus Elternsicht zu analysieren und Variablen zu explorieren, die diese elterlichen Erwartungen beeinflussen.

**Zusatzmaterial online:**

Zusätzliche Informationen sind in der Online-Version dieses Artikels (10.1007/s11618-023-01149-9) enthalten.

## Abstract (en)

In light of the Covid-19-related school lockdowns in Germany in 2020 schools, families and the students were faced with the major challenge to continue instruction at home. This paper examines the expectations of the parents that their children will experience school-related problems as a result to the lockdown-induced homeschooling within the next six months. For our explorative analysis, we choose a nonlinear regression approach. In the course of this, we introduce nonlinear models and highlight their added value compared to methods commonly used in empirical educational research. For the analysis we combine data from the National Educational Panel Study (NEPS) with additional data sources like the COVID-19-Dashboard of the Robert-Koch-Institut (RKI). Our results show that parental expectations of future school problems were particularly prevalent among those parents whose children had low reading competencies and low diligence as an aspect of school effort. In addition, we find a relationship between a lower occupational status (ISEI) and higher parental expectations of school-related problems. Furthermore, parents’ short-term and long-term concerns about Covid-19 show a positive association, making school problems more likely in the eyes of the parents. The purpose of this paper, in addition to applying and explaining nonlinear models for the first time in empirical educational research, is to analyze expectations regarding problems of homeschooling in the first lockdown from a parents’ perspective and to explore variables that influence these parental expectations.

## Einleitung

Am 13. März 2020 beschlossen die Länder der Bundesrepublik Deutschland, die Schulen aufgrund der Coronapandemie bundesweit bis auf Weiteres zu schließen. Damit sahen sich die Bildungsinstitutionen flächendeckend mit der Herausforderung konfrontiert, die Interaktion mit den Schüler*innen in den digitalen Raum zu verlagern und bislang nur wenig genutzte Möglichkeiten des digitalen Unterrichtens einzusetzen. Studien, die das Homeschooling in Deutschland untersuchen, zeigen, dass die neue Unterrichtssituation nachteilige Folgen nach sich gezogen hat (Anger et al. 2021). Leistungsschwächere (Grewenig et al. 2021; Schult et al. 2022) und sozioökonomisch benachteiligte (Huber et al. 2020) Schüler*innen waren besonders betroffen und zusammen mit denjenigen, die zuvor in der Schule zusätzliche Förderung benötigten, laufen sie durch das Homeschooling Gefahr, weitere Leistungsdefizite aufzubauen (Goldan et al. 2020). Ähnliche Zusammenhänge finden sich auch international. Eine Studie aus den Niederlanden stellt einen starken Lernrückgang bei Schüler*innen während des Lockdowns fest, wobei ein Zusammenhang zwischen einer niedrigeren sozialen Herkunft und dem Zurechtkommen mit dem Homeschooling besteht (Engzell et al. 2021).

In dem vorliegenden Beitrag wird der Zusammenhang zwischen den Erwartungen der Eltern, dass innerhalb der kommenden sechs Monate bei ihren Kindern Schulprobleme auftreten werden als abhängige Variable und unabhängigen Variablen aus den folgenden vier Themenfeldern untersucht: (1) der soziale Hintergrund sowie (2) die Ressourcen und Vorkenntnisse der Schüler*innen, die sich in bestehenden Studien bereits andeuten. Weiterhin wird geprüft, (3) inwiefern der dauerhafte Aufenthalt zu Hause und die eingeschränkten Freizeitmöglichkeiten dem erweiterten sozialen Umfeld in der Wohnumgebung der Schüler*innen Bedeutung verleiht. Zudem wird untersucht, (4) welche Zusammenhänge von der Pandemie selbst ausgehen, z. B. durch Ängste oder durch Veränderungen der beruflichen Situation von Eltern.

Mit dem Ziel, statistische Erkenntnisse über die Auswirkungen des Homeschoolings für die Schüler*innen in Deutschland zu gewinnen, wird gezeigt, wie Daten des Nationalen Bildungspanels (NEPS; Blossfeld und Roßbach 2019) genutzt werden können, um in Kombination mit externen Datenquellen eine Datengrundlage für eine explorative Fragestellung zu einem neuen Themenfeld zu schaffen. Weiterhin wird in interdisziplinärer Zusammenarbeit von empirischer Bildungsforschung und angewandter Statistik präsentiert, wie Variablen unterschiedlicher Skalenniveaus mithilfe von generalisierten additiven Modellen modelliert werden können, so dass auch Rückschlüsse auf nichtlineare Zusammenhänge möglich werden. Auf diese Weise können in einem explorativen Setting auch solche Assoziationen statistisch robust geschätzt werden, die den in der Bildungsforschung etablierten Verfahren, z. B. der Modellierung linearer Zusammenhänge in Regressionsanalysen, verborgen bleiben oder verzerrt dargestellt werden.

## Theoretischer Rahmen und aktuelle Forschungslage

Im Folgenden werden vor dem Hintergrund des aktuellen Forschungsstands und unter Bezugnahme auf theoretische Modelle die Forschungsfragen hergeleitet. Dabei wird insbesondere auf die elterlichen Erwartungen im Zusammenhang mit den Leistungen der Kinder, auf die Bedeutung des sozialen Umfelds, der Kompetenzen der Schüler*innen, der Wichtigkeit der Nachbarschaft und der familialen Belastungen durch die Coronapandemie für den Umgang mit dem Homeschooling eingegangen. Zusätzlich wird die Methodik der bestehenden Studien betrachtet, um die Bedarfe für das analytische Vorgehen der vorliegenden Untersuchung abzuleiten.

### Elterliche Erwartungen von Schulproblemen

Aufgrund der Verlagerung des Unterrichts von der Schule in das eigene Zuhause waren Eltern durch ihre Unterstützungsfunktion stärker in das Lernen ihrer Kinder involviert und teilweise sogar als einzige Personen für die Durchführung des Homeschoolings verantwortlich (Bujard et al. 2021). Entsprechend wurde der Einfluss der Eltern auf die Leistungen der Kinder intensiviert (Bujard et al. 2021; Hillmayr et al. 2021). Dass elterliche Handlungen bereits vor der Pandemie eine große Rolle für den Leistungserfolg von Kindern spielten, zeigen Grgic und Bayer (2015), die die Interaktion zwischen Eltern und Kindern untersuchten und feststellten, dass das aktuelle schulische Befinden seitens der Kinder von den Eltern beeinflusst wird und dass diese auf die schulischen Selbsteinschätzungen der Kinder einwirken. Auch Täschner et al. (2021) erörtern in einem Second-Order-Review den Zusammenhang zwischen Elternbeteiligung und schulischem Erfolg und kommen zu dem Ergebnis, dass neben den elterlichen Bildungserwartungen auch eine lernförderliche Umgebung zu Hause für die Leistungen der Kinder bedeutsam ist.

Zusätzlich zu der Betreuung ihrer Kinder mussten die Eltern während des Lockdowns ihren eigenen beruflichen Verpflichtungen nachkommen, die sich ihrerseits durch die Pandemie verändert haben können, z. B. durch die Arbeit im Homeoffice. Insbesondere Mütter, welche in den meisten Fällen hauptsächlich die Unterstützung des Homeschoolings in der Pandemie übernahmen (Bujard et al. 2021), erlebten dadurch eine Verschmelzung von Arbeits- und Familienzeit und damit eine Verlagerung der Arbeit bis in die Nacht hinein (Beham-Rabanser et al. 2022), da sie die wegfallenden Tagesabläufe und Strukturen abfangen mussten.

Es ist anzunehmen, dass nicht alle Eltern der Herausforderung, ihre Kinder in schulischen Belangen vermehrt zu unterstützen und sie im Homeschooling anzuleiten, in gleichem Maße gewachsen waren und dass sich dies auf die Leistungen der Kinder auswirkt. Als diejenigen Personen, die das schulische Lernen im Homeschooling am engsten begleiten und angesichts des aus ihrer Sicht wenig ausgeprägten Kontakts zwischen den Schüler*innen und den Lehrkräften bzw. Schulen (Helm et al. 2021), sollten die Eltern am ehesten in der Lage sein, die zu erwartenden Konsequenzen auf die Leistungen der Schüler*innen einzuschätzen. In der NEPS-Sonderbefragung zu Corona wurden die Eltern gebeten anzugeben, für wie wahrscheinlich sie es halten, dass ihre Kinder im Laufe der nächsten sechs Monate Schulprobleme entwickeln. Dabei handelt es sich um die zum Zeitpunkt dieser Studie bestmögliche Proxy-Variable im Rahmen des NEPS, um die Auswirkung des Homeschoolings auf die schulischen Leistungen von Schüler*innen zu betrachten. Sie wird in der vorliegenden Untersuchung als abhängige Variable auf Zusammenhänge mit den im folgenden dargestellten Einflussfaktoren hin analysiert.

### Ressourcen des Lernens der Schüler*innen

Neben dem Einfluss der Eltern trugen unterschiedliche Vorkenntnisse und Kompetenzen dazu bei, dass Schüler*innen nicht gleichermaßen in der Lage waren, das Homeschooling erfolgreich zu bewältigen. Leistungsstärkere Schüler*innen mit einer höheren Lesekompetenz und einer höheren Anstrengungsbereitschaft waren motivierter und damit auch erfolgreicher (Bujard et al. 2021; Lockl et al. 2021), während leistungsschwächere Schüler*innen weniger erfolgreich mitarbeiten konnten. Damit drohte eine Intensivierung bestehender Leistungsunterschiede (Lockl et al. 2021). Auch Schüler*innen, die bereits vor dem Homeschooling zusätzliche Hilfe benötigten, liefen Gefahr, die Leistungsdefizite weiter auszubauen (Goldan et al. 2020). Des Weiteren ging der Erwerb der Lesekompetenz im Homeschooling stark zurück. Ludewig et al. (2022) finden einen starken Lernrückgang bei Viertklässler*innen im Zusammenhang mit der Coronapandemie im Vergleich zu den Kompetenzen von Viertklässler*innen im Jahre 2016.

Das von Helmke (2012) vorgeschlagene Angebot-Nutzungs-Modell stellt Unterricht als ein Angebot dar, das von Lehrkräften zur Verfügung gestellt und von Schüler*innen genutzt wird. Dabei erzielen die Schüler*innen Erträge in Form von Lernergebnissen, die von verschiedenen äußeren Faktoren beeinflusst werden. Züchner und Jäkel (2021) erweitern das Angebot-Nutzungs-Modell im Zusammenhang mit der Pandemie und dem damit verbundenen Homeschooling und zeigen, dass der Fokus auf selbstständigem, schülerzentriertem Arbeiten liegt, welches eher mit Hausaufgaben als mit schulischem Lernen zu vergleichen ist (Huber et al. 2020). Insgesamt zeichnet sich ab, dass die Lehrkräfte deutlich weniger in das Homeschooling involviert waren als die Eltern erwarteten und den Schüler*innen selbst eine größere Verantwortung für das Lernen übertragen wurde (vgl. Grewenig et al. 2021; Steinmayr und Christiansen 2020). Für den Lernerfolg der Schüler*innen werden in der Pandemie-Situation damit neben den von Helmke (2012) beschriebenen persönlichen Voraussetzungen für das Lernen, wie Vorkenntnisse, Lernmotivation und Anstrengungsbereitschaft auch das Selbst- und Zeitmanagement zu wichtigen Ressourcen (Züchner und Jäkel 2021).

### Soziale Herkunft

Nicht alle Eltern konnten die von den Kindern im Homeschooling benötigte Unterstützung in gleicher Weise leisten: Je nach Studie gaben 24 bis 63 % der Eltern an, weniger als eine Stunde pro Tag für die Unterstützung aufzuwenden (Cordes 2020; Heller und Zügel 2020; Helm et al. 2021; Wildemann und Hosenfeld 2020). Dies mag individuell unterschiedliche Gründe haben, Studien verweisen jedoch auf einen systematischen Zusammenhang zwischen dem Lernerfolg und dem sozialen Status der Eltern (Engzell et al. 2021). Damit läuft das coronabedingte Homeschooling Gefahr, die ohnehin in Deutschland deutlich ausgeprägten Leistungsunterschiede aufgrund des sozialen Hintergrunds weiter zu verstärken (Eickelmann und Drossel 2020; forsa 2020; Hußmann et al. 2017; Stubbe et al. 2020; Weis et al. 2019).

In der Terminologie von Boudon (1974) dürfte die neue Rolle der Eltern insbesondere primären Herkunftseffekten Vorschub leisten. Hierbei handelt es sich um die Effekte des sozialen Status der Eltern, die sich direkt auf die Schulleistungen der Kinder auswirken. Eltern höheren sozialen Status können zusätzliche Ressourcen zur Förderung des Lernerfolgs ihrer Kinder zur Verfügung stellen (z. B. privat bezahlte Nachhilfe (Schneider 2006)). Eltern mit höherem Bildungsniveau sind zudem eher in der Lage und sehen eher die Notwendigkeit, ihre Kinder bei schulischen Aufgaben zu unterstützen (Bol 2020; Lochner 2020). So standen beispielsweise auch schon vor der Coronapandemie Hausaufgaben in der Kritik, da die elterliche Unterstützung in diesem Bereich deutlich mit dem Lernerfolg der Kinder korreliert (Hillmayr et al. 2021) und die Schere zwischen leistungsstarken und leistungsschwachen Schüler*innen vergrößert (Hagenauer und Oberwimmer 2019). Da das Lernen in der Homeschooling-Situation der Arbeit an Hausaufgaben ähnelt (Züchner und Jäkel 2021) und die häusliche Unterstützung der Eltern erfordert, kann davon ausgegangen werden, dass auch hier deutliche primäre Herkunftseffekte zu verzeichnen sein werden.

Viele Studien zu Zusammenhängen der sozialen Herkunft folgen den theoretischen Annahmen von Bourdieu (1983) und analysieren die Effekte des ökonomischen, kulturellen und sozialen Kapitals der Eltern auf die Schulleistungen. Wie einem Working Paper (Sari et al. 2021) zur Situation während der ersten Schulschließung in Deutschland zu entnehmen ist, sind für die Frage, inwiefern Eltern ihren Kindern bei schulischen Aufgaben helfen können, insbesondere das kulturelle Kapital – in Form von Büchern zu Hause und der Bildung der Eltern – sowie das soziale Kapital – in Form des elterlichen sozialen Netzwerks – von Bedeutung. Zusätzlich zu Effekten des sozialen Hintergrunds bestehen auch nachteilige Effekte für Familien mit Migrationshintergrund: Kinder von Eltern nicht-deutscher Herkunftssprache hatten eher Schwierigkeiten mit der Bewältigung der Aufgaben im Homeschooling (Züchner und Jäkel 2021).

### Veränderte Bedeutung der unmittelbaren Nachbarschaft

Nicht nur die Schulen, sondern auch die meisten Einrichtungen des öffentlichen und kulturellen Lebens, z. B. Vereine, Musikschulen und Kinos wurden geschlossen und die Nutzung von Spielplätzen wurde untersagt (§1 Abs. 3 Nds. GVBl. 10/2020). Dadurch fielen die meisten Möglichkeiten der Freizeitgestaltung für Kinder und Jugendliche weg. Viele litten laut Aussagen ihrer Eltern darunter (Wildemann und Hosenfeld 2020). Durch diese eingeschränkten Möglichkeiten der Freizeitgestaltung lässt sich annehmen, dass die Wohnsituation sowie die unmittelbare Nachbarschaft der Familien für das tägliche Leben während der Pandemie und das Homeschooling an Bedeutung gewonnen haben.

Bereits ohne die Einschränkungen einer Pandemie ist die Nachbarschaft für die (schulische) Entwicklung von Kindern und Jugendlichen von Bedeutung: Verschiedene theoretische Ansätze wie etwa zu Rollenbildern und kollektiven Sozialisationsprozessen (Bandura 1973; Bandura und Walters 1963) legen dar, wie Kinder und Jugendliche von der Nachbarschaft, in der sie leben, beeinflusst werden können. Studien zeigen beispielsweise, dass ein höherer sozialer Status in der Nachbarschaft Effekte auf das Verhalten von Jugendlichen hat, z. B. im Hinblick auf einen vorzeitigen Schulabbruch (Crane 1991). Einzelne Studien für Deutschland bzw. deutsche Städte verdeutlichen, dass die soziale Segregation in Großstädten insbesondere Kinder betrifft (Helbig und Jähnen 2018) und von einer privilegierteren Nachbarschaft positive Effekte auf die Kompetenzentwicklung von Kindern ausgehen (Helbig 2010). Eine Studie aus Köln verweist weiterhin darauf, dass eine höhere Arbeitslosenquote zu einer geringeren Verfügbarkeit von Rollenvorbildern führt, was wiederum das Arbeitslosigkeitsrisiko für Jugendliche steigert (Nonnenmacher 2009, 2013).

Auch wenn es sich beim Homeschooling im Frühjahr 2020 in Deutschland um einen begrenzten Zeitraum handelte, ist davon auszugehen, dass die unmittelbare Nachbarschaft für das Familienleben an Bedeutung gewinnt, da die Möglichkeiten zum Kontakt außerhalb dieser durch Homeoffice und Homeschooling eingeschränkt waren. Dabei ist zu erwarten, dass sich eine Zusammensetzung der Nachbarschaft aus sozial höher gestellten Personen vorteilhaft auswirkt, während sozial deprivierte Nachbarschaften nachteilige Effekte erzeugen. Begründen lässt sich dies z. B. durch die theoretischen Annahmen zu sozialen Netzwerken: In deprivierten Wohngegenden leben Familien mehr oder weniger isoliert von sozialen Netzwerken (Jencks und Mayer 1990), in sozial besser gestellten Wohnvierteln, z. B. mit einem höheren Anteil Akademiker*innen, dürfte es hingegen leichter sein, insbesondere für schulische Belange hilfreiche Kontakte zu knüpfen. Eine Studie von del Bello et al. (2015) mit Daten US-amerikanischer Jugendlicher verweist zudem auf *peer*-Effekte im Zusammenhang mit Lernergebnissen: Sowohl in der Schule als auch in der Nachbarschaft haben *peers* einen Effekt auf das Abschneiden in schulischen Tests. Zwar ergeben die Analysen, dass die Interaktion mit *peers* in der Schule wichtiger ist, es scheint jedoch plausibel, dass sich dies in einer Pandemiesitutation ändert, da die Schulen geschlossen und Schulkamerad*innen nur online verfügbar sind. Auch wenn Treffen für Kinder und Jugendliche in Deutschland im ersten Lockdown zahlenmäßig begrenzt waren, könnte es von Bedeutung sein, wie viele ihrer *peers* sie ohne weite Wege in ihrer direkten Nachbarschaft erreichen konnten und welche Möglichkeiten zur Freizeitgestaltung ihnen angesichts der geschlossenen Freizeiteinrichtungen, z. B. im Garten der Eltern, zur Verfügung standen.

### Betroffenheit der Familie durch die Coronapandemie

Neben den durch die Coronapandemie ausgelösten Veränderungen des öffentlichen und privaten Lebens waren viele Familien auch direkt von Corona betroffen, z. B. durch eine eigene Corona-Erkrankung oder durch Sorgen, die sich aufgrund veränderter beruflicher und finanzieller Aspekte für die Zukunft ergaben. Höhere regionale Inzidenzen könnten die Einschränkungen und Ängste der Eltern dabei intensivieren. Nicht jedes Elternteil wird zudem die Möglichkeit gehabt haben, im Homeoffice zu arbeiten, so dass die Betreuungssituation für Kinder schwieriger wurde. Ausgehend vom *Family Stress Model* (Conger et al. 1994) kann angenommen werden, dass insbesondere Veränderungen der beruflichen und finanziellen Situation für die Eltern mit Stress verbunden sind, welcher sich auf deren Umgang mit ihren Kindern auswirkt und zu einer emotionalen Belastung seitens dieser führen kann (Zinn und Bayer 2021). Nach Heintz-Martin und Langmeyer (2020), die im Rahmen des *Family Stress Models* die ökonomische Situation in Familien untersuchen, haben finanzielle Einschränkungen und Armut, wie sie sich auch im Rahmen der Coronapandemie für zahlreiche Familien ergeben haben, einen Einfluss auf das Wohlbefinden des Kindes. Je nach Betroffenheit der Familie durch die Pandemie könnten diese besonders ausgeprägt sein und sich entsprechend auch auf das Zurechtkommen mit dem Homeschooling auswirken und letztendlich dazu führen, dass Schulprobleme auftreten bzw. im Rahmen der verfügbaren Datengrundlage durch den Proxy der elterlichen Erwartung prognostiziert werden.

### Theoretisches Analysemodell

**Figure 1:**
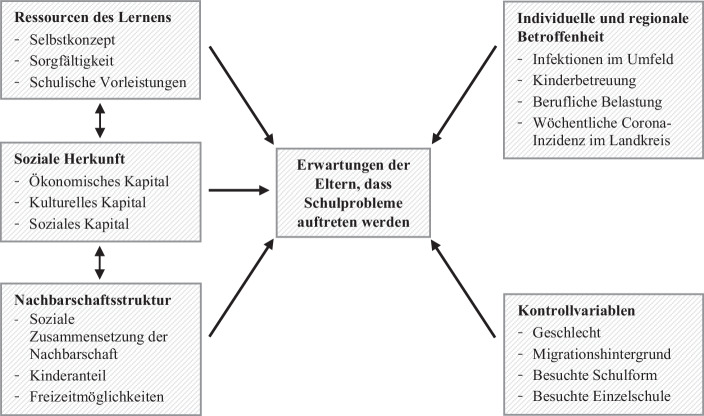

Ziel dieses Beitrags ist es, in explorativer Herangehensweise zu untersuchen, welche Zusammenhänge die oben skizzierten Hintergrund- und Kontextmerkmale mit der Bewältigung der Schulschließungen im Frühjahr 2020 aufweisen. Konkret soll gezeigt werden, inwiefern sich aus Sicht der Eltern aus dem Homeschooling in den folgenden sechs Monaten Schulprobleme für ihre Kinder entwickeln werden und inwiefern diese Erwartung mit den Kompetenzen der Kinder, deren sozialer Herkunft, ihrer Wohnumgebung bzw. Nachbarschaft, der individuellen Betroffenheit der Familien durch die Coronapandemie sowie der regionalen Inzidenzen zusammenhängen. Den Analysen wird das folgende theoretische Analysemodell zugrunde gelegt (s. Abb. 1).

Zusätzlich wird diese Fragestellung genutzt, um aufzuzeigen, welche methodischen Möglichkeiten das NEPS bietet. Zum einen wird auf die Analysepotenziale eingegangen, die sich aus der Kombination des NEPS mit zusätzlichen externen Datenquellen ergeben. Zum anderen werden statistische Analyseverfahren angewendet und erläutert, die neue Erkenntnisse für die empirische Bildungsforschung ermöglichen, bislang in der Disziplin aber wenig Verbreitung finden, wie die semi-parametrische Regression.

## Methoden

### Daten

Die folgenden Analysen basieren auf den NEPS-Daten der Startkohorte 2 (SC2) und den Daten der NEPS-Zusatzerhebungen zur Coronapandemie (NEPS-C) (Blossfeld und Roßbach 2019; NEPS-Netzwerk 2020; NEPS-Netzwerk 2021). Die Startkohorte 2 startete 2011 mit Kindern, die zu diesem Zeitpunkt 4 Jahre alt waren und einen Kindergarten besuchten. Die Kinder wurden hinsichtlich ihrer Kompetenzen in verschiedenen Bereichen getestet. Zusätzlich wurden ihre Eltern, Erzieher*innen und Einrichtungsleitungen per Telefoninterview befragt. Im Jahr 2013 wechselten die Kinder in die Grundschule und die Stichprobe wurde um zusätzliche Erstklässler*innen aufgestockt. Die in den vorliegenden Analysen genutzten Daten stammen zu großen Teilen aus der aktuellen Befragungswelle 9, in der Schüler*innen die 7. Jahrgangsstufe besuchten. NEPS‑C wurde als freiwillige Zusatzbefragung außerhalb des ursprünglichen Befragungsturnus des NEPS in den Startkohorten 2 bis 6 durchgeführt. Zwischen dem 13. Mai und dem 22. Juni 2020 nahmen insgesamt *n* = 1587 Eltern der Startkohorte 2 teil, die den Ausgangspunkt der folgenden Analysen darstellen. Nach Ausschluss von Förderschüler*innen und unplausiblen Extremwerten bleiben *n* = 1556 Fälle für die folgenden Analysen.

Die NEPS-Daten werden um zwei Arten von Regionaldaten ergänzt: (1) durch Informationen zu sozialen Kontextmerkmalen in der Nachbarschaft der Familien aus dem in Verbindung mit dem NEPS verfügbaren microm-Datensatz (Schönberger und Koberg 2016) und (2) durch Corona-Fallzahlen auf Kreisebene aus dem RKI COVID-19-Dashboard (Robert Koch-Institut 2021). Hierzu wurden die tagesaktuellen RKI Corona-Fallzahlen einzelner Landkreise von esri über den ArcGIS Hub (https://hub.arcgis.com/) mittels HTTP-Programming automatisiert heruntergeladen und für das Matching mit den NEPS-Daten um die Gemeindeschlüssel der Landkreise auf NUTS3-Ebene ergänzt. Zur Ermittlung der wöchentlichen Corona-Inzidenzen pro 100.000 Einwohner, die in die Analysen eingehen, wurden die aktuellen Bevölkerungszahlen der Landkreise hinzugezogen (Destatis 2021). Um den Datenschutzanforderung des NEPS gerecht zu werden und eine eindeutige Zuordnung der Befragten zu den Landkreisen über die Inzidenzwerte auszuschließen, wurden die Inzidenzen in Intervallen mit einer Schrittweite von 5 kategorisiert und Mittelwerte der resultierenden Kategorien gebildet.

### Operationalisierung

Im Folgenden wird die Operationalisierung der ausgewählten Merkmale auf Grundlage des Forschungsstandes und einem anschließend durchgeführten Modellwahlverfahren für die Regressionsanalysen vorgestellt.

#### Erwartete Schulprobleme aus Elternsicht

Die abhängige Variable der vorliegenden Untersuchung, *Erwartete Schulprobleme aus Elternsicht (a. E.),* ist die Einschätzung der Eltern, mit welcher Wahrscheinlichkeit sie auf einer Skala von 0 bis 100 % aufgrund der aktuellen Situation für die kommenden sechs Monate Schulprobleme für ihre Kinder erwarten.

#### Ressourcen des Lernens

Die Vorleistungen der Schüler*innen werden anhand der *wle-*Scores (*weighted maximum likelihood estimates*) aus den NEPS-Leistungstests in der 7. Klasse in den Fächern Lesen, Mathematik und Naturwissenschaften in das Analysemodell aufgenommen. Die *wle-*Scores sind Punktschätzer für individuelle Kompetenzen, wobei ein *wle-*Score von 0 einer durchschnittlichen Kompetenz entspricht. Werte über 0 entsprechen einer überdurchschnittlichen Kompetenz, Werte unter 0 einer unterdurchschnittlichen (für eine genauere Erläuterung der *wle-*Scores siehe Pohl und Carstensen 2012). Weiterhin werden das Selbstkonzept und die Anstrengungsbereitschaft betrachtet. Das Selbstkonzept wird anhand von drei Items und die Anstrengungsbereitschaft anhand von vier Items operationalisiert. Beide wurden mithilfe einer Faktorenanalyse (Bestimmung der Anzahl der Faktoren durch Eigenwert-Kriterium, Extraktion der Faktoren mit obliquer Rotation) zu Regressionsscores zusammengefasst. Die Analysen ergaben einen Faktor für das *schulische Selbstkonzept* (Beispielitem: „In den meisten Schulfächern lerne ich schnell.“, Originalskala von 1 „trifft gar nicht zu“ bis 4 „trifft voll und ganz zu“; Cronbachs Alpha = 0,85) und zwei Faktoren für die Anstrengungsbereitschaft, die die *Sorgfältigkeit* (Beispielitem: „Ich erledige alle Aufgaben mit großer Sorgfalt“, Originalskala von 1 „stimme gar nicht zu“ bis 4 „stimme völlig zu“; Cronbachs Alpha = 0,71) und das *Durchhaltevermögen* (Beispielitem: „Ich gebe schnell auf, wenn mir etwas schwer fällt“, Originalskala von 1 „stimme gar nicht zu“ bis 4 „stimme völlig zu“; Cronbachs Alpha = 0,71) der Schüler*innen abbilden.

#### Soziale Herkunft

Entsprechend der Definition von Bourdieu (1983) wird die soziale Herkunft der Schüler*innen anhand des ökonomischen, kulturellen und sozialen Kapitals der Familie operationalisiert. Das ökonomische Kapital wird in den Analysen durch das monatliche Haushaltseinkommen der Eltern und durch den *International Socio-economic Index of Occupational Status 2008 *(ISEI, auf einer Skala von 12 bis 89) der Eltern einbezogen, der als Indikator für deren beruflichen Status dient (Ganzeboom 2010). In die Analysen geht der jeweils höhere der beiden ISEI-Werte der Eltern als Variable *Höchster beruflicher Status der Eltern* (*ISEI*) ein. Das kulturelle Kapital wird anhand des höchsten Bildungsabschlusses der Eltern analysiert, der zu der Variable *Hochschulabschluss der Eltern* zusammengefasst wurde. Diese gibt Auskunft darüber, ob mindestens ein Elternteil über einen Hochschulabschluss verfügt. Weiterhin wird die Variable *Anzahl der Bücher zu Hause* berücksichtigt (Kategorien: „weniger als 100 Bücher“, „101 bis 200 Bücher“, „201 bis 500 Bücher“ und „mehr als 500 Bücher“). Das soziale Kapital wird nach Coleman (1988) als Kapital innerhalb und außerhalb der Familie definiert. Für das Kapital innerhalb der Familie wird die *Unterstützungszeit der Eltern für schulische Aufgaben (vor Corona)*, aufgenommen. Soziales Kapital außerhalb der Familie wird durch die Angaben der Eltern zum Positionsgenerator operationalisiert (Lin und Dumin 1986) und geht als Variable *Anzahl Berufe im sozialen Netzwerk der Eltern* ein. Dabei geben Eltern an, wie viele von 13 Berufen sich in ihrem sozialen Netzwerk finden. Ein diverses Netzwerk wird als die potenzielle Verfügbarkeit von mehr Ressourcen und daher mehr verfügbares Kapital gedeutet (Lin und Dumin 1986).

#### Nachbarschaftsstruktur

Um potenzielle Zusammenhänge zwischen den *erwarteten Schulproblemen a. E.* und der Wohnumgebung der Familie zu berücksichtigen, werden die *Arbeitslosenquote im Viertel *und die *Akademikerquote im Häuserblock *als Indikatoren für die soziale Zusammensetzung der Nachbarschaft einbezogen. Die *Arbeitslosenquote *ist der prozentuale Anteil der Erwerbslosen an der Gesamtzahl der zivilen Erwerbspersonen im Viertel (PLZ-8-Gebiete). Die *Akademikerquote *gibt Auskunft über den Anteil der Akademiker*innen an allen Personen über 25 Jahren im Häuserblock (ca. 5–8 Häuser) des Wohnorts (Kategorien: 1 „bis unter 2 %“, 2 „von 2 % bis unter 3 %“, 3 „von 3 % bis unter 4 %“, 4 „von 4 % bis unter 5 %“, 5 „von 5 % bis unter 7,5 %“, 6 „von 7,5 % bis unter 10 %“, 7 „von 10 % bis unter 12,5 %“, 8 „von 12,5 % bis unter 25 %“, 9 „über 25 %“). Zusätzlich geht die Variable *dominantes Geo-Milieu* als weiterer Indikator für die Zusammensetzung der Nachbarschaft (Ausprägungen: „adaptiv-pragmatisch“, „bürgerliche Mitte“, „expeditiv“, „hedonistisch“, „konservativ-etabliert“, „liberal-intellektuell“, „Performer“, „prekär“, „sozial-ökologisch“, „traditionell“) in die Analysen ein. Zur Ermittlung möglicher *peer*-Zusammenhänge wird der Anteil der Kinder im Straßenzug der Befragten einbezogen (Codierung: 1 „niedriger Kinderanteil“ bis 9 „hoher Kinderanteil“). Die *Wahrscheinlichkeit eigener Garten, *also die Wahrscheinlichkeit für das Vorhandensein eines eigenen Gartens, geht als Indikator für erweiterte Aufenthalts- und Freizeitmöglichkeiten für die Familien in die Analysen ein (Codierung: 1 „niedrige Wahrscheinlichkeit“ bis 9 „hohe Wahrscheinlichkeit“) (vgl. Schönberger und Koberg 2016).

#### Individuelle und regionale Betroffenheit durch Corona

Im Hinblick auf die individuelle Betroffenheit durch Corona wird analysiert, ob die Eltern selbst oder in ihrem Freundes- und Bekanntenkreis im Befragungszeitraum eine Corona-Infektion erlebt haben (*Corona-Infektionen im persönlichen Umfeld*). Weiterhin werden die *kurz-* und *langfristigen Sorgen* der Eltern im Hinblick auf die Corona-Situation betrachtet. Aus insgesamt fünf Items wurden per Faktorenanalyse (siehe oben) zwei Skalen ermittelt, die als Regressionsscores angeben, inwieweit Eltern sich kurz- (Beispielitem: „Wenn Sie an die Zukunft denken, wie stark machen Sie sich Sorgen darüber, dass das Gesundheitssystem überlastet wird?“, Originalskala: 0 „gar nicht“ bis 10 „sehr große Sorgen“; Cronbachs Alpha: 0,57) oder langfristig (Beispielitem: „Wenn Sie an die Zukunft denken, wie stark machen Sie sich Sorgen darüber, dass der Unterschied zwischen Arm und Reich größer wird?“, Originalskala: 0 „gar nicht“ bis 10 „sehr große Sorgen“; Cronbachs Alpha = 0,84) um die Folgen der Coronapandemie sorgen. Weiterhin wird analysiert, welche Zusammenhänge sich durch die berufliche Belastung, d. h., dass Eltern in einem systemrelevanten Beruf arbeiten (*Systemrelevanter Beruf*) und keine Möglichkeit hatten, im Homeoffice zu arbeiten (*Kein Homeoffice möglich*) sowie der Notwendigkeit, die Kinderbetreuung selbst zu übernehmen (*Kinderbetreuung durch die Eltern*), ergeben. Anhand der Inzidenz-Daten des RKI (s. Abschn. 3.1) wird zudem die unmittelbare Bedrohung durch das Coronavirus über die wöchentliche Inzidenz pro 100.000 Einwohner im Landkreis der Befragten analysiert.

#### Kontrollvariablen

In allen Analysen wird die *besuchte Schulform* (Kategorien: 1 „Hauptschule“, 2 „Realschule“, 3 „Schule mit mehreren Bildungsgängen“, 4 „Gymnasium“) der Schüler*innen sowie zusätzlich die Zugehörigkeit zu einer Einzelschule (*Besuchte Einzelschule*) berücksichtigt. Weiterhin werden ein möglicher *Migrationshintergrund* (mindestens ein Elternteil im Ausland geboren) sowie das biologische *Geschlecht* der Schüler*innen als Kontrollvariablen in die Analysen aufgenommen.

### Statistische Modellierung

Abweichend von der konventionellen Verwendung klassischer linearer Modelle zur Schätzung von statistischen Zusammenhängen in der empirischen Bildungsforschung schlagen wir die Nutzung eines nichtlinearen Modellierungsansatzes für die oben beschriebenen explorativen Fragestellungen vor. Im Folgenden wird diese Erweiterung des statistischen Repertoires skizziert. Zunächst wird die Modellklasse nichtlinearer additiver Modelle vorgestellt und mit konventionellen linearen Modellen kontrastiert. Anschließend wird die zugrunde liegende Methodik von Basisfunktionen und Splines sowie die Interpretationsmöglichkeiten der resultierenden Schätzung für die abhängige Variable erläutert. Abschließend wird die Erweiterung nichtlinearer additiver Modelle auf den multivariaten Kontext und die Variablenselektion mittels des Akaike Informationskriteriums (im Folgenden AIC, Akaike 1983) erläutert.

#### Lineare und Nichtlineare Additive Modelle

Die nach wie vor dominante Form der statistischen Modellierung in vielen Anwendungsbereichen – so auch in der empirischen Bildungsforschung – ist die lineare Regressionsanalyse. Hierbei wird eine Zielvariable *y* mit unabhängigen Variablen $x_{1}{,}\ldots {,}x_{k}$ in den folgenden funktionalen Zusammenhang gesetzt:

1 $$\left(y\right)=\beta _{0}+\beta _{1}x_{1}+\beta _{2}x_{2}+\ldots +\beta _{k}x_{k}+\epsilon$$

Dem linearen Modell wird in den folgenden Analysen eine nichtlineare Modellierung der folgenden Form entgegengesetzt:

2 $$(y)=f_{1}\left(x_{1}\right)+f_{2}\left(x_{2}\right)+\ldots +f_{k}\left(x_{k}\right)+\epsilon$$

Im Rahmen dieser Modellierung sind die funktionalen Zusammenhänge $f_{1}{,}\ldots {,}f_{k}$ zwischen den jeweiligen Variablen $x_{1}{,}\ldots {,}x_{k}$ und der abhängigen Variable nicht mehr zwangsläufig linear, sondern folgen einem potenziell nichtlinearen Verlauf. Der Einfachheit halber werden an dieser Stelle gängige Erweiterungen wie der Einsatz von Link-Funktionen auf die abhängige Variable, funktionale Formen einzelner unabhängiger Variablen durch Polynome oder die Berücksichtigung von Abhängigkeitsstrukturen der Störterme vernachlässigt (für eine detaillierte Darstellung siehe Fahrmeir et al. 2007). Um den Unterschied zwischen den beiden Modellierungsformen zu illustrieren, betrachten wir den univariaten Zusammenhang zwischen der unabhängigen Variable *Lesekompetenz* und der abhängigen Variable *erwartete Schulprobleme a. E.* im Rahmen der in Abschn. 3.3.4 beschriebenen Modellierung.

**Figure 2:**
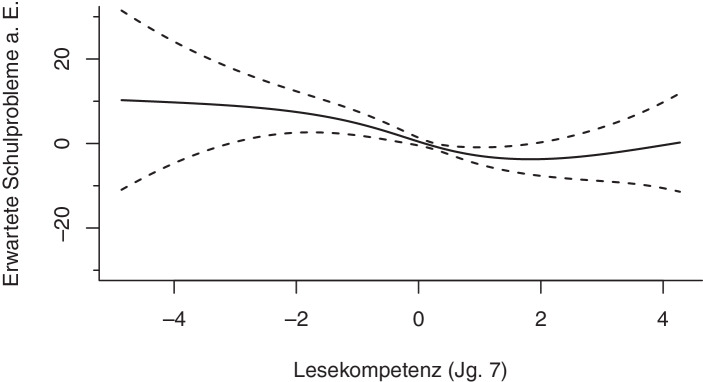

Wie Abb. 2 zu entnehmen ist, folgt eine flexiblere Modellierung gemäß der Gl. 2 für den Zusammenhang zwischen der *Lesekompetenz *und den *erwarteten Schulproblemen a. E.* keinem linearen Verlauf, welcher durch eine Gerade mit einer konstanten Steigung über den gesamten Verlauf der X‑Achse dargestellt werden würde. Vielmehr werden sehr unterschiedliche Steigungsparameter über den Verlauf des Kovariablenraums sichtbar. Während beispielsweise zwischen −4 und −2 die Steigung vergleichsweise gering ist, fällt sie zwischen −1 und 1 deutlich steiler ab. Zwischen 2 und 4 wird sogar einen Anstieg der *erwarteten Schulprobleme a. E.* bei zunehmender *Lesekompetenz *ersichtlich. Anstelle einer vorgegebenen funktionalen Annahme über die Form des globalen Zusammenhangs zwischen den Variablen tritt somit eine flexiblere Form der Modellierung, die im Folgenden mit dem konventionellen Ansatz der linearen Regression kontrastiert wird.

#### Globale und lokale lineare Modellierung

**Figure 3:**
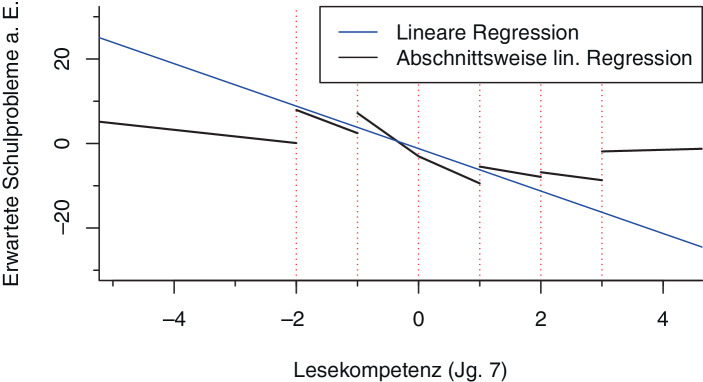

Im Fall einer linearen Modellierung ergibt sich auf Basis der inhärenten Modellannahmen ein linearer Zusammenhang zwischen der *Lesekompetenz *und *erwarteten Schulproblemen a. E.*, welcher durch eine Gerade mit konstanter Steigung gekennzeichnet ist. In Abb. 3 ist dieser lineare Zusammenhang graphisch dargestellt, wobei für eine bessere Vergleichbarkeit mit der nichtlinearen Regression hier ausschließlich dieser eine Zusammenhang linear modelliert wird und für die verbleibenden Variablen die in Abschn. 3.3.3 beschriebene nichtlineare additive Modellierungsform genutzt wird.

Die zu beobachtenden Abweichungen von der rigiden Form der linearen Modellierung und der nichtlinearen Modellierung wird durch eine lokale Schätzung des funktionalen Zusammenhangs anstelle einer globalen Schätzung ermöglicht. Um diesen zugrunde liegenden Unterschied zwischen der linearen Modellierung und nichtlinearen Modellierung zu illustrieren, wird zunächst eine vereinfachte Vorstufe der Spline-Regression betrachtet.

In Abb. 3 wird außerdem eine abschnittsweise Schätzung des funktionalen Zusammenhangs dargestellt. Hierfür wurde der betrachtete Definitionsbereich der *Lesekompetenz *in sieben Intervalle unterteilt und für jedes Intervall eine eigenständige lineare Schätzung durchgeführt. Durch diese abschnittsweise Schätzung wird die globale lineare Schätzung nun durch lokale Schätzungen des Zusammenhanges auf den jeweiligen Intervallen ersetzt. Wie in der nichtlinearen Schätzung in Abb. 2 ist auch hier zu sehen, dass sich die Steigungen auf den jeweiligen Intervallen unterscheiden und somit von einem variierenden Zusammenhang zwischen der *Lesekompetenz *und den *erwarteten Schulproblemen a. E.* ausgegangen werden kann. Aufgrund der disjunkten Natur der einzelnen lokalen Schätzungen lassen sich neben variierenden Gradienten über den Variablenverlauf unnatürliche Sprünge an den Intervallgrenzen beobachten. Um diese Sprünge im Rahmen lokaler Schätzungen zu verhindern und einen hinreichend glatten Funktionsverlauf sicherzustellen, wird die einfache intervallweise Schätzung, wie im folgenden Abschnitt dargestellt, erweitert.

#### Nichtlineare Modellierung mittels Splines

**Figure 4:**
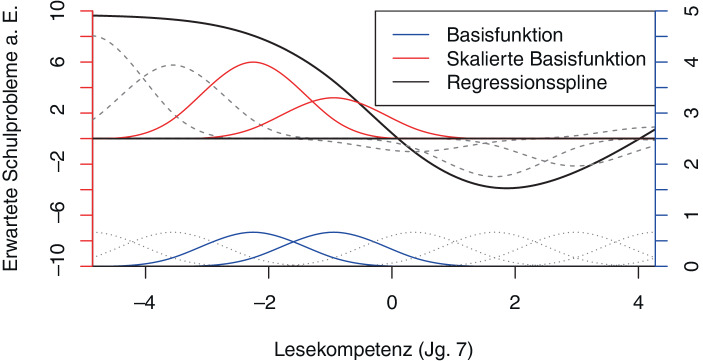

Analog zum obigen Beispiel wird der Kovariablenraum in Teilmengen unterteilt, wobei üblicherweise ca. 20 Knotenpunkte genutzt werden, welche die Teilmengen separieren. Die daraus resultierenden Teilmengen der Daten werden genutzt, um den Zusammenhang zwischen den Variablen lokal zu schätzen. Die Schätzung läuft dabei über eine Vielzahl von Basisfunktionen, welche in Abb. 4 am unteren Rand dargestellt sind.

Jede dieser Basisfunktionen nutzt den Wert der unabhängigen Variablen als Funktionsargument, um auf Grundlage der Nähe zum Zentrum des jeweiligen Intervalls die Bedeutung der jeweiligen Beobachtung zu gewichten und sie entsprechend dieses Gewichtes in die lokale Schätzung einfließen zu lassen. Das entsprechende Gewicht ist für das Gros der Beobachtungen gleich Null, sodass die jeweilige lokale Schätzung nur aufgrund einer überschaubaren Teilmenge der Beobachtungen erfolgt und lokale Eigenheiten effektiv abgebildet werden können. Im Gegensatz zu dem obigen vereinfachten Ansatz der abschnittsweisen Schätzung ist darauf hinzuweisen, dass sich die Intervalle einiger der Basisfunktionen überlappen, wie beispielsweise die beiden in blau dargestellten Basisfunktionen. Folglich sind die zugrunde liegenden Datenmengen für die einzelnen lokalen Schätzungen nicht vollständig disjunkt – was zu einem stetigen Funktionsverlauf ohne Sprünge führt. Ferner ist die Form der Basisfunktionen typischerweise nicht linear, was konzeptionelle und numerische Vorteile mit sich bringt, wobei unterschiedliche Formen der Basisfunktionen zur Verfügung stehen (für Details siehe Wood 2017).

Für die Schätzung des durch die schwarze Linie dargestellten globalen Zusammenhangs *f*_*j*_(*x*_*j*_) zwischen der *Lesekompetenz *und den *erwarteten Schulproblemen a. E.* müssen die Basisfunktionen über zu schätzende Koeffizienten skaliert werden. Die skalierten Basisfunktionen sind in der mittleren Ebene in Rot dargestellt. Die Schätzung der Koeffizienten kann grundsätzlich über verschiedene gängige Verfahren wie kleinste Quadrate oder *Maximum Likelihood* erfolgen. Es existieren verschiedene Formen von Basisfunktionen und damit Splines, wobei hier Thin Plate Regressionsplines aus dem R‑Paket mgcv (Wood 2017) genutzt werden. Anschließend werden für jeden Punkt im Definitionsbereich die skalierten Basisfunktionen aufaddiert. Die daraus resultierende Funktion *f*(*x*) ist somit ein Resultat mehrerer Schätzungen, welche in unterschiedlichen Gewichtungen die lokalen Eigenheiten der Daten widerspiegelt. Durch einen Bestrafungsterm werden Sprünge im Funktionsverlauf verhindert und extreme Schwankungen im Funktionsverlauf aufgrund von einer Überanpassung an die Daten vermieden. Die Stärke des Bestrafungsterms wird datengetrieben durch ein Restricted Maximum Likelihood (REML) Verfahren geschätzt (für Details siehe Wood 2017).

#### Die Interpretation von Splines

Die Interpretation des Zusammenhanges in linearen Modellen kann in der Regel direkt über die Parameter erfolgen. Im Fall des Illustrationsbeispiels wurde ein Steigungsparameter $\beta$= −3,75 geschätzt, sodass gemäß dem Modell zwischen der *Lesekompetenz *und den *erwarteten Schulproblemen a. E.* ein negativer linearer Zusammenhang besteht, d. h. bei einer zusätzlichen *Lesekompetenz *von einer Einheit (*wle*-Score) fällt die Wahrscheinlichkeit, mit der Eltern Schulprobleme erwarten, um 3,75 %.

Die Interpretation des nichtlinearen Modells hingegen ist üblicherweise nicht direkt über die Parameter möglich, da im Regelfall für die Schätzung des globalen Zusammenhangs 20 Parameter geschätzt werden, welche überlappend in die Schätzung des lokalen Funktionsverlaufs an verschiedenen Stellen einfließen. Zur Interpretation kann der Funktionsverlauf visuell inspiziert und bei Bedarf die Ableitung bzw. lokale Steigung an unterschiedlichen Punkten berechnet werden. Beispielsweise kann durch die Bestimmung des Funktionswertes an den jeweiligen Eckpunkten auf dem Intervall von −2 bis 0 eine durchschnittliche Steigung von −3,45 berechnet werden, während sie im Intervall von +2 bis +4 den Wert 1,17 annimmt. Dabei ergibt sich die Auswahl der Intervalle, für welche die Steigungen bestimmt werden, aus der zugrundeliegenden Fragestellung. Insbesondere die visuelle Darstellung erlaubt im Regelfall bereits einen umfassenden Einblick in die Natur des Zusammenhangs.

Hinsichtlich der Signifikanz eines Zusammenhangs bei nichtlinearer Modellierung erfolgt die Betrachtung üblicherweise auf globaler Ebene. Aufbauend auf den Standardfehlern der zugrunde liegenden Koeffizienten kann für die geschätzte Funktion *f*_*j*_(*x*_*j*_) ein Konfidenzband für ein gegebenes Signifikanz-Niveau geschätzt werden, um den Raum der wahrscheinlichen Verläufe der Funktion abzuschätzen – ähnlich wie dies im Fall des linearen Modells mithilfe des Konfidenzintervalls für den Steigungsparameter möglich ist.

Das entsprechende 95 %-Konfidenzband für die *Lesekompetenz *ist in Abb. 2 abgebildet. Es ist zu sehen, dass die Schätzunsicherheit in Bereichen mit wenig Beobachtungen am linken und rechten Rand besonders hoch ist.

Ebenso lässt sich auf Grundlage der Standardfehler der einzelnen Koeffizienten und den gängigen Annahmen der *p*-Wert für die Hypothese berechnen, dass über den Verlauf des betrachteten Kovariablenraums eine Abweichung von einem konstanten Verlauf zu beobachten ist. Das entsprechende Analogon hierzu im linearen Modell wäre der *p*-Wert für den Steigungsparameter der Gerade.

Wie in Tab. 2 im Ergebnisteil dargelegt, ist der *p*-Wert für die *Lesekompetenz *0,01, sodass von einem signifikanten Zusammenhang zwischen den zwei Variablen ausgegangen werden kann.

Zuletzt ist noch eine Betrachtung der effektiven Freiheitsgrade (*effective degrees of freedom*, im Folgenden *edf*) aufschlussreich. Die effektiven Freiheitsgrade können, ähnlich wie konventionelle Freiheitsgrade, als Maß für die Komplexität des Modells bzw. des einzelnen geschätzten Effektes gesehen werden. Bei linearen Modellen besteht ein direkter Zusammenhang zwischen der Anzahl der geschätzten Parameter und der durch das Modell verbrauchten Freiheitsgerade. So erfordert ein einfacher linearer Zusammenhang die Schätzung eines Parameters und verbraucht somit einen Freiheitsgrad. Spline-artige Modellierungen können bei hinreichender Nichtlinearität der Daten hingegen mehr und durch den Bestrafungsterm auch nicht ganzzahlige Freiheitsgrade verbrauchen. Im Falle der *Lesekompetenz *beobachten wir 2,87 effektive Freiheitsgrade. Dieses Resultat impliziert, dass der geschätzte Zusammenhang deutlich komplexer ist als ein linearer Zusammenhang, welcher ein *edf*-Maß nahe 1 liefern würde.

#### Multivariate nichtlineare Modellierung, Variablenselektion und Erweiterungen

Hinsichtlich der Erweiterungen auf einen multivariaten Kontext wird normalerweise ein additiver Zusammenhang zwischen der abhängigen Variable und den unabhängigen Variablen unterstellt, wenngleich bei Bedarf z. B. über Tensor-Product-Splines höherdimensionale nichtlineare (Hyper‑)Oberflächen modelliert werden können (siehe Wood 2017). Für die explorative Analyse wird entsprechend der gängigen Annahme einer additiven Relation gefolgt, sodass die Modellierung gemäß der Gl. 2 erfolgt.

Um zu bestimmen, welche Variablen in die nichtlineare Modellierung aufgenommen werden sollen, wird eine Backward-Selektion über das AIC eingesetzt. Der Grundgedanke dieses weit verbreiteten Gütemaß ist es, jenes Modell zu wählen, welches eine adäquate Balance zwischen der internen Modellierungsgüte gegenüber den Stichprobendaten und der externen Modellierungsgüte bzw. der Modellkomplexität aufweist. Auf Grundlage dieses Auswahlkriteriums wird im Rahmen der Backward-Selektion zu Beginn ein Modell mit allen theoretisch als relevant erachteten und praktisch verfügbaren Variablen evaluiert und mit Modellen verglichen, welche eine Variable weniger aufweisen. Anschließend werden sukzessive weitere potenzielle Variablenreduktionen evaluiert, bis jegliche weitere Reduktion zu einer Verschlechterung des AIC führt. Mittels dieses Verfahrens kann somit aus einer häufig unübersichtlichen Menge denkbarer Modelle datengetrieben ein favorisiertes Modell ausgewählt werden. Wenngleich die Nutzung von Modellselektionsverfahren auch im Kontext linearer Modellierung oftmals sinnvoll ist, ist sie in Anbetracht der größeren Komplexität nichtlinearer Modellierung von noch größerer Relevanz.

Die in den folgenden Analysen genutzte REML basierte Schätzung ermöglicht einen aus statistischer Sicht eleganteren Umgang mit fehlenden Werten als die konventionelle Nutzung imputierter Werte. In dem von Wood (2017) vorgeschlagenen Verfahren werden fehlende Werte über zufällige Zusammenhänge repräsentiert, sodass die durch fehlende Werte entstehende Variabilität der Schätzung direkt und ohne potenziell verfälschende (mehrfache) Imputationen berücksichtigt werden kann. Außer für jene Werte, für welche eine deterministische Imputation auf Grundlage früher Wellen gerechtfertigt war – wie z. B. bei zeitinvariaten Variablen – wird bei der Schätzung die skizzierte Methodik verwendet.

Der Grundgedanke der nichtlinearen Modellierung kann nicht nur auf metrische Variablen angewandt werden. Der Ansatz von lokaler Schätzung mit Bestrafungstermen lässt sich auch für diskrete Variablen adaptieren. Im Gegensatz zur konventionellen Verwendung unrestringierter Dummy-Kombinationen für solche Variablen werden somit üblicherweise weniger Freiheitsgrade verbraucht, was stabilere Schätzergebnisse nach sich zieht und Ordnungsstrukturen in den Kovariablen berücksichtigt. Eine solche Modellierung wird für die Variablen *Besuchte Schulform, Anzahl der Bücher zu Hause* und *Akademikeranteil im Häuserblock* verwendet. Für dichotome Variablen und nominale Variablen ohne Ordnungsstruktur ist eine nichtlineare Modellierung hingegen nicht sinnvoll, sodass für diese Variablen eine konventionelle Dummy-Kodierung verwendet wird.

#### Limitationen nichtlinearer Modellierung

Die nichtlineare Modellierung steht im Kontrast zu der konventionellen Modellierung mit linearer Regression, welche eine globale Schätzung des Funktionsverlaufes und rigide Annahmen über die funktionale Form des Zusammenhangs zwischen der abhängigen und unabhängigen Variable vorgibt. Die rigide Form der linearen Modellierung mit einer einzelnen globalen Parameterschätzung hat den nicht zu vernachlässigenden Vorteil, dass sie konzeptionell und bzgl. des Rechenaufwands weniger anspruchsvoll ist als die Darstellung über Splines.

Insbesondere bei der Interpretation des Zusammenhangs zwischen zwei Variablen, welche auf Grundlage eines einzelnen Parameters erfolgen kann, hat die simplifizierende Annahme der Linearität offensichtliche Vorteile. Es gilt schlichtweg zu konstatieren, dass die Interpretation über einen einzelnen konstanten Parameter deutlich einfacher und im Zweifel auch eindrücklicher ist, als die Interpretation auf Grundlage eines potenziell komplexen nichtlinearen Zusammenhanges welcher üblicherweise eine bildliche Darstellung erfordert.

Des Weiteren sollte nicht verschwiegen werden, dass der zugrunde liegende Rechenaufwand bei der nichtlinearen Modellierung um ein Vielfaches höher ist als für die lineare Modellierung. Wenngleich moderne Computer im Regelfall ausreichend Rechenleistung mitbringen, ist die Wahrscheinlichkeit technischer Probleme bei der Schätzung (z. B. arithmetischer Unterlauf o. ä.) naturgemäß höher. Angesichts der im Regelfall aber überschaubaren Komplexität von Anwendungen im Bereich der empirischen Bildungsforschung ist diese Problematik aus unserer Sicht zu vernachlässigen.

Entsprechend sollte in der empirischen Bildungsforschung die Nutzung von den anspruchsvolleren nichtlinearen Modellierungsmethoden insbesondere dann in Erwägung gezogen werden, wenn davon auszugehen ist, dass die Natur des Zusammenhanges zwischen den jeweiligen Kovariablen keiner einfachen funktionalen Form folgt oder eine explorative Analyse ohne vorab spezifizierte inhärente Einschränkungen – wie sie der linearen Regression zugrunde liegen – durchgeführt werden soll.

Ein weiterer oftmals nicht unerheblicher Vorteil der nichtlinearen Modellierung über Splines ist die bereits skizzierte lokale Schätzung des Zusammenhangs anstelle einer globalen Schätzung über den gesamten Kovariablenraum. Dies ist insbesondere dann von Vorteil, wenn Beobachtungen an den Rändern des Kovariablenraums, welche eine hohe Hebelwirkung auf die Schätzung entfalten könnten, Gefahr laufen, als Ausreißer die Schätzung in überbordendem Maße zu beeinflussen.

Ob sich der rechnerische und interpretative Mehraufwand der Anwendung von nichtlinearer Modellierung anstelle von rein linearer Modellierung lohnt, hängt in erster Linie davon ab, ob die Forschenden von der sowohl einschränkenden als auch einhegenden Annahme der Linearität der einzelnen additiven Zusammenhänge abrücken möchten oder nicht.

## Ergebnisse

Im Folgenden werden die Ergebnisse der durchgeführten Analysen vorgestellt. Mithilfe der oben beschriebenen AIC-basierten Modellwahl wurden die folgenden Variablen in das Analysemodell einbezogen: Mathematikkompetenz der Schüler*innen (*in der 7. Jahrgangsstufe*), *Lesekompetenz* (*Jg. 7*), *Naturwissenschaftliche Kompetenz* (*Jg. 7*), Schulisches Selbstkonzept der Schüler*innen, Sorgfältigkeit der Schüler*innen, Höchster beruflicher Status der Eltern (*ISEI*), *Hochschulabschluss der Eltern, Monatliches Haushaltseinkommen der Eltern, Anzahl der Bücher zu Hause, Unterstützungszeit der Eltern für schulische Aufgaben (vor Corona), Anzahl Berufe im sozialen Netzwerk der Eltern, Arbeitslosenquote im Viertel, Akademikeranteil im Häuserblock, Kinderanteil im Straßenzug, Wahrscheinlichkeit eigener Garten, Mittlere wöchentliche Inzidenz im Landkreis zum Befragungszeitpunkt, Kein Homeoffice möglich, Kinderbetreuung durch die Eltern, Kurzfristige Sorgen durch Corona, Langfristige Sorgen durch Corona, Besuchte Schulform, Besuchte Einzelschule* und *Geschlecht*. Nicht ausgewählt wurden: *Dominantes Geo-Milieu*, *Corona-Infektionen im persönlichen Umfeld*, *Systemrelevanter Beruf der Eltern*, *Migrationshintergrund*, Durchhaltevermögen der Schüler*innen.

Zunächst wird auf die deskriptive Ausprägung der betrachteten Variablen eingegangen. Anschließend werden die Ergebnisse der nichtlinearen Spline-Regression dargestellt und zuletzt die Ergebnisse der Spline-Regression mit denen der einfachen multiplen linearen Regression verglichen. Dabei wird in der Darstellung auf Merkmale eingegangen, die einen signifikanten Zusammenhang zeigen. Darstellungen zu allen weiteren Merkmalen finden sich im Anhang.

### Deskriptive Ergebnisse

**Figure 5:**
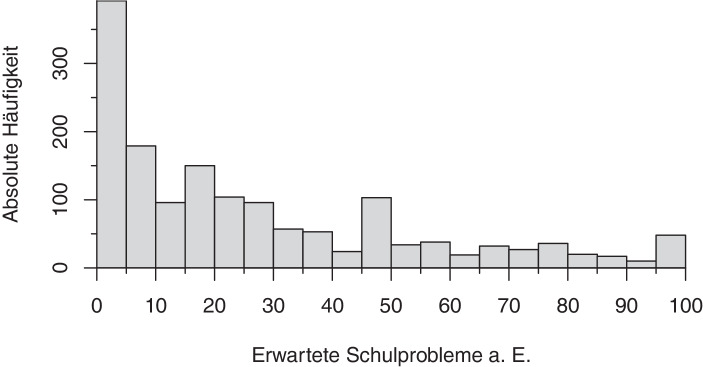

Die Wahrscheinlichkeit, mit der Eltern Schulprobleme aufgrund der Schulschließungen erwarten, ist in der betrachteten Stichprobe rechtsschief verteilt (s. Abb. 5). 16 % der Eltern geben an, mit einer Wahrscheinlichkeit von null Prozent Schulprobleme aufgrund der Schulschließungen zu erwarten. 76 % rechnen mit einer Wahrscheinlichkeit von weniger als 50 % mit zukünftigen Schulproblemen ihrer Kinder.

Tab. 1 zeigt die Mittelwerte und Standardabweichungen bzw. prozentualen Anteile der in die Regressionsanalysen einbezogenen Merkmale. Die Ressourcen des Lernens der Schüler*innen sind entsprechend des methodischen Vorgehens (Mittelwerte für alle betrachteten Scores = 0^1^) durchschnittlich ausgeprägt. Hinsichtlich der sozialen Herkunft ist die hier betrachtete Stichprobe etwas positiv verschoben: Das ökonomische Kapital der Eltern (ISEI), das kulturelle Kapital in Form von Bildung und das soziale Kapital gemessen durch den Positionsgenerator fallen überdurchschnittlich aus. In der Nachbarschaft der Familien liegt die Arbeitslosenquote bei etwa 5 %, der Kinderanteil fällt etwas überdurchschnittlich aus, die Wahrscheinlichkeit, dass ein eigener Garten vorhanden ist, ist leicht erhöht. Die regionale Bedrohung durch Corona fällt – im Vergleich zu später gemessenen Inzidenzwerten in der Pandemie – mit ca. 4 Fällen pro 100.000 Einwohner*innen im Landkreis der Befragten zum Befragungszeitpunkt gering aus. Nichtsdestotrotz berichten 37 % der Befragten von Corona-Fällen in ihrem Familien‑, Freundes- und Bekanntenkreis. Hinsichtlich der beruflichen Belastung zeigt sich, dass etwa 45 % der befragten Eltern in einem systemrelevanten Beruf arbeiten. 49 % konnten in der hier betrachteten Phase der Pandemie nicht im Homeoffice arbeiten, 61 % mussten die Betreuung ihrer Kinder während der Schulschließungen selbst übernehmen. Die Schüler*innen der untersuchten Stichprobe sind zu 46 % männlich, 30 % von ihnen haben einen Migrationshintergrund und besuchen am häufigsten ein Gymnasium (68 %).

**Table 1:** 

	*M*^*a*^	*SD*^*a*^	Anteil^b^
**Ressourcen des Lernens**			
Mathematikkompetenz (Jg. 7) (wle-Score)	0,22	1,62	–
Lesekompetenz (Jg. 7) (wle-Score)	0,23	1,30	–
Naturwissenschaftl. Kompetenz (Jg. 7) (wle-Score)	0,18	0,94	–
Schulisches Selbstkonzept (Regressionsscore)	0,06	0,94	–
Sorgfältigkeit (Regressionsscore)	0,00	0,86	–
**Soziale Herkunft**			
Höchster beruflicher Status der Eltern (ISEI) (12–89)	58,89	19,05	–
Hochschulabschluss der Eltern: ja	–	–	0,57
Monatliches Haushaltseinkommen der Eltern	3064,48	1607,85	–
Anzahl der Bücher zu Hause	–	–	–
*Weniger als 100*	–	–	0,27
*101 bis 200*	–	–	0,20
*201 bis 500*	–	–	0,21
*Mehr als 500*	–	–	0,22
Unterstützungszeit für schulische Aufgaben (vor Corona) (min./Woche)	78,89	87,94	–
Anzahl Berufe im soz. Netzwerk der Eltern (0–13)	8,53	2,25	–
**Merkmale der Nachbarschaft**			
Arbeitslosenquote im Viertel (in %)	5,48	4,35	–
Akademikeranteil im Häuserblock			
*Unter 2 %*	–	–	0,07
*2–3 %*	–	–	0,12
*3–4 %*	–	–	0,12
*4–5 %*	–	–	0,09
*5–7,5 %*	–	–	0,15
*7,5–10 %*	–	–	0,09
*10–12,5 %*	–	–	0,05
*12,5–25 %*	–	–	0,16
*Über 25 %*	–	–	0,14
Kinderanteil im Straßenzug (1 = niedrig – 9 = hoch)	6,05	2,49	–
Wahrscheinlichkeit eigener Garten (1 = niedrig – 9 = hoch)	6,07	2,30	–
**Individuelle und regionale Betroffenheit durch Corona**			
Mittlere wöchentl. Inzidenz im Landkreis zum Befragungszpkt. (pro 100.000 Einw.)	3,89	3,92	–
Kein Homeoffice möglich: ja	–	–	0,49
Kinderbetreuung durch die Eltern: ja	–	–	0,62
Kurzfristige Sorgen durch Corona (Regressionsscore)	0,01	0,94	–
Langfristige Sorgen durch Corona (Regressionsscore)	0,01	0,81	–
**Kontrollvariablen**			
Besuchte Schulform			
*Hauptschule*	–	–	0,02
*Realschule*	–	–	0,12
*Schule mit mehreren Bildungsgängen*	–	–	0,18
*Gymnasium*	–	–	0,68
Geschlecht: männlich	–	–	0,46

*n* = 1556

^*a*^ Mittelwerte und Standardabweichungen für (quasi-)metrische Variablen

^*b*^ Anteile für kategoriale Variablen

### Ergebnisse der nichtlinearen Regressionsanalyse

Im Folgenden werden die Ergebnisse der nichtlinearen Regression tabellarisch (s. Tab. 2) und signifikante Effekte im Sinne der Interpretierbarkeit der funktionalen Form des Zusammenhangs zusätzlich graphisch dargestellt (s. Abb. 2 und 6, 7, 8, 9, 10). Insgesamt zeigt die nichtlineare Regressionsanalyse, dass die Wahrscheinlichkeit, mit der die befragten Eltern Schulprobleme aufgrund des coronabedingten Homeschoolings erwarten, vor allem von den Ressourcen des Lernens ihrer Kinder und ihrer individuellen Betroffenheit durch die Coronapandemie abhängt (s. Tab. 2).

**Figure 6:**
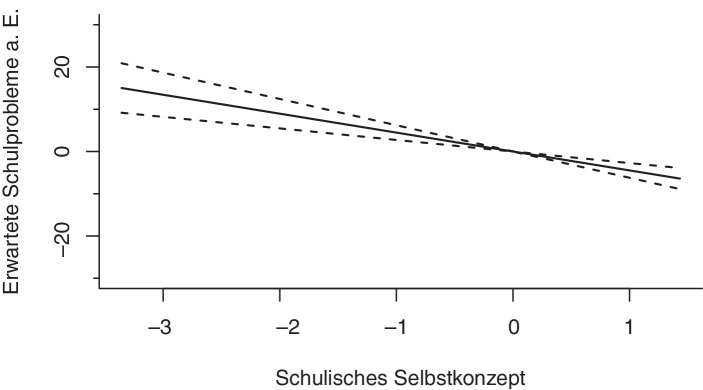

**Figure 7:**
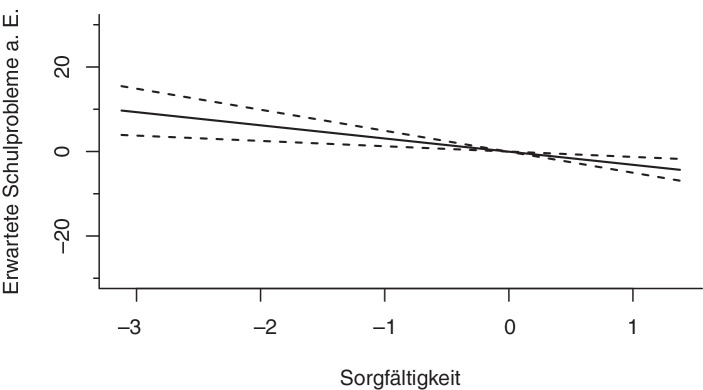

**Figure 8:**
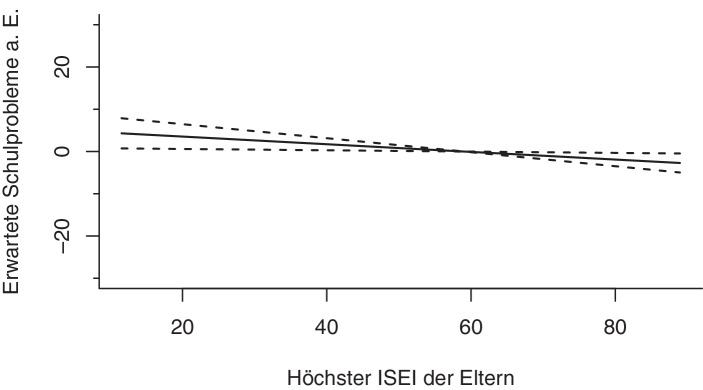

**Figure 9:**
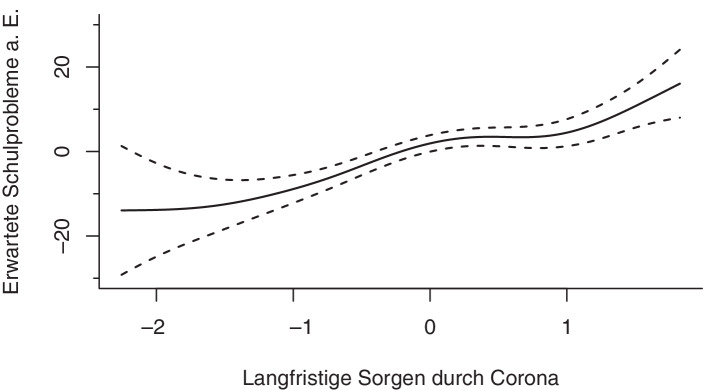

**Figure 10:**
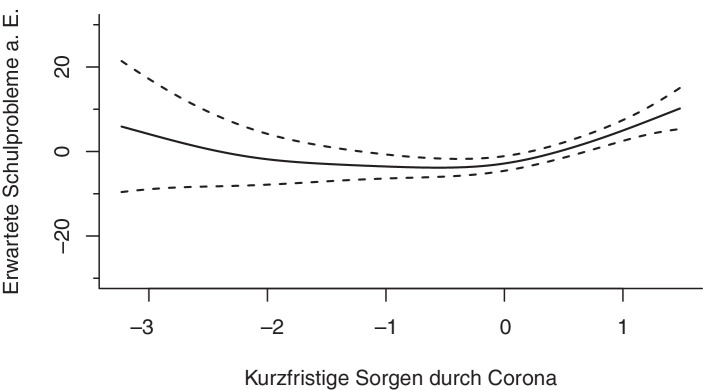

**Table 2:** 

	*β* ^a^	*edf* ^b^	*p*
y‑Achsenabschnitt	24,86	–	0,00
**Ressourcen des Lernens**			
Mathematikkompetenz (Jg. 7)	–	1,00	0,38
Lesekompetenz (Jg. 7)	–	2,87	0,01
Naturwissenschaftl. Kompetenz (Jg. 7)	–	1,00	0,06
Schulisches Selbstkonzept	–	1,00	0,00
Sorgfältigkeit	–	1,00	0,00
**Soziale Herkunft**			
Höchster beruflicher Status der Eltern (ISEI)	–	1,00	0,02
Hochschulabschluss der Eltern	−2,44	–	0,09
Monatliches Haushaltseinkommen der Eltern	–	1,00	0,17
Anzahl der Bücher zu Hause	–	0,25	0,27
Unterstützungszeit für schulische Aufgaben (vor Corona)	–	1,00	0,30
Anzahl Berufe im soz. Netzwerk der Eltern	–	1,00	0,72
**Merkmale der Nachbarschaft**			
Arbeitslosenquote im Viertel	–	2,16	0,29
Akademikeranteil im Häuserblock	–	0,00	0,61
Kinderanteil im Straßenzug	–	1,50	0,39
Wahrscheinlichkeit eigener Garten	–	2,87	0,10
**Betroffenheit durch Corona**			
Mittlere wöchentl. Inzidenz im Landkreis zum Befragungszpkt.	–	1,00	0,20
Kein Homeoffice möglich	2,26	–	0,10
Kinderbetreuung durch die Eltern	2,06	–	0,14
Kurzfristige Sorgen durch Corona	–	3,22	0,00
Langfristige Sorgen durch Corona	–	4,24	0,00
**Kontrollvariablen**			
Besuchte Schulform	–	0,95	0,06
Männliches Geschlecht	2,40	–	0,08

*n* = 1556

^*a*^ Geschätzte Regressionskoeffizienten β für dichotome Variablen

^b^ Effective degrees of freedom für ordinale und (quasi-)metrische Variablen

#### Ressourcen des Lernens

Das nichtlineare Regressionsmodell zeigt für die Ressourcen des Lernens signifikante Zusammenhänge für das schulische Selbstkonzept und die Sorgfältigkeit. Die effective degrees of freedom von *edf* = 1,00 (*Selbstkonzept*) und *edf* = 1,00 (*Sorgfältigkeit*) spiegeln die Linearität des Zusammenhangs wider: Je höher das Selbstkonzept und je höher die Sorgfältigkeit eine*r Schüler*in, umso geringer ist die Wahrscheinlichkeit, mit der Eltern Schulprobleme erwarten (s. Abb. 6 und 7). Die Vorleistungen der Schüler*innen im Bereich Lesen zeigen ebenfalls einen signifikanten Zusammenhang mit den erwarteten Schulproblemen aus Elternsicht. Während die befragten Eltern von Schüler*innen im unteren Kompetenzbereich mit einer um ungefähr zehn Prozentpunkte erhöhten Wahrscheinlichkeit Schulprobleme erwarten, lässt sich bei durchschnittlichen Kompetenzen zunächst ein Abfall der erwarteten Schulprobleme a. E. erkennen, der sich im oberen Kompetenzbereich wieder leicht umkehrt (s. Abb. 2 in Abschn. 3.3.1).

#### Soziale Herkunft

Die Analysen zeigen weiterhin einen signifikanten, annähernd linearen Zusammenhang (*edf* = 1,00) zwischen dem ökonomischen Kapital (*Höchster beruflicher Status der Eltern* (*ISEI*) mit den erwarteten Schulproblemen a. E. Je höher das ökonomische Kapital, mit desto geringerer Wahrscheinlichkeit erwarten Eltern Schulprobleme (s. Abb. 8).

#### Merkmale der Nachbarschaft

Für die Merkmale der Nachbarschaft zeigen sich keine signifikanten Zusammenhänge mit den erwarteten Schulproblemen a. E.

#### Individuelle und regionale Betroffenheit durch Corona

Die individuelle Betroffenheit durch die Coronapandemie in Form der mit Corona verbundenen kurzfristigen und langfristigen Sorgen der Eltern zeigen signifikante Zusammenhänge mit den erwarteten Schulproblemen aus Elternsicht. Obwohl der Zusammenhang zwischen den langfristigen Sorgen der Eltern und den erwarteten Schulproblemen nicht linear ist (*edf* = 4,24), zeigt sich ein klarer Trend: Je stärker die mit Corona verbundenen langfristigen Sorgen der Eltern sind, mit desto höherer Wahrscheinlichkeit erwarten sie Schulprobleme (Abb. 9). Auch bei einer überdurchschnittlichen Ausprägung der kurzfristigen Sorgen durch Corona zeigt sich ein Anstieg der erwarteten Schulprobleme aus Elternsicht. Der geschätzte Spline zeigt zudem, dass Eltern, deren mit Corona verbundenen kurzfristige Sorgen stark unterdurchschnittlich ausgeprägt sind, mit einer leicht erhöhten Wahrscheinlichkeit Schulprobleme erwarten, wobei einschränkend zu sagen ist, dass die Schätzung der erwarteten Schulprobleme in diesem Bereich wenig stabil ist, wie an der Breite der Konfidenzbänder zu erkennen ist (Abb. 10).

#### Kontrollvariablen

Hinsichtlich der betrachteten Kontrollvariablen zeigen sich keine signifikanten Zusammenhänge mit den *erwarteten Schulproblemen a. E.*

### Vergleich der nichtlinearen mit der multiplen linearen Regressionsanalyse

Tab. 3 zeigt die Ergebnisse der multiplen linearen Regressionsanalyse: In Übereinstimmung mit der nichtlinearen Spline-Regression zeigen sich in der multiplen linearen Regression für die erwarteten Schulprobleme aus Elternsicht signifikante Zusammenhänge der Sorgfältigkeit und des schulischen Selbstkonzepts der Schüler*innen sowie der kurz- und langfristigen Sorgen der Eltern. Zusätzliche signifikante Zusammenhänge bestehen zwischen der Mathematikkompetenz der Schüler*innen sowie der Möglichkeit der Eltern, im Homeoffice zu arbeiten. Die in der nichtlinearen Regressionsanalyse signifikanten Zusammenhänge der Lesekompetenz der Schüler*innen und des elterlichen Berufsstatus (ISEI) auf die erwarteten Schulprobleme sind in der multiplen linearen Regression nur noch als nicht signifikanter Trend erkennbar.

**Table 3:** 

	β	*p*
y‑Achsenabschnitt	36,21	0,00
**Ressourcen des Lernens**		
Mathematikkompetenz (Jg. 7)	2,79	0,02
Lesekompetenz (Jg. 7)	−3,75	0,09
Naturwissenschaftl. Kompetenz (Jg. 7)	−1,90	0,35
Schulisches Selbstkonzept	−4,01	0,00
Sorgfältigkeit	−3,46	0,00
**Soziale Herkunft**		
Höchster beruflicher Status der Eltern (ISEI)	−0,08	0,06
Hochschulabschluss der Eltern	−2,82	0,10
Monatliches Haushaltseinkommen der Eltern	0,00	0,11
Anzahl der Bücher zu Hause		
*101 bis 200*	−0,37	0,88
*201 bis 500*	−1,33	0,52
*Mehr als 500*	−1,36	0,44
Unterstützungszeit für schulische Aufgaben (vor Corona)	0,01	0,34
Anzahl Berufe im soz. Netzwerk der Eltern	−0,13	0,68
**Merkmale der Nachbarschaft**		
Arbeitslosenquote im Viertel	0,20	0,24
Akademikeranteil im Häuserblock		
*2–3 %*	0,53	0,87
*3–4 %*	−5,09	0,13
*4–5 %*	−1,02	0,76
*5–7,5 %*	0,98	0,78
*7,5–10 %*	0,69	0,83
*10–12,5 %*	−1,88	0,60
*12,5–25 %*	−2,66	0,47
*Über 25 %*	0,18	0,96
Kinderanteil im Straßenzug	−0,27	0,31
Wahrscheinlichkeit eigener Garten	0,18	0,59
**Betroffenheit durch Corona**		
Mittlere wöchentl. Inzidenz im Landkreis zum Befragungszpkt.	−0,15	0,38
Kein Homeoffice möglich	3,04	0,04
Kinderbetreuung durch die Eltern	1,06	0,50
Kurzfristige Sorgen durch Corona	3,70	0,00
Langfristige Sorgen durch Corona	7,07	0,00
**Kontrollvariablen**		
Besuchte Schulform		
*Realschule*	9,43	0,13
*Schule mit mehreren Bildungsgängen*	1,07	0,68
*Gymnasium*	3,32	0,12
Männliches Geschlecht	0,71	0,63

*n* = 1556

Insbesondere beim Zusammenhang zwischen den erwarteten Schulproblemen a. E. und der Lesekompetenz vor der Pandemie lässt sich somit festhalten, dass der empirische Zusammenhang zum einen Nichtlinearitäten in der Form aufweist, welche durch die Anzahl von 2,87 effektiven Freiheitsgraden ausgewiesen werden. Zum anderen ist festzuhalten, dass im Zwischenspiel zwischen der nichtlinearen empirischen Datenstruktur und der Linearitätsannahme bei der multiplen linearen Regressionsanalyse auch der *p*-Wert über die gängige 5 %-Schwelle fällt (*p* = 0,01 im nichtlinearen Modell vs. *p* = 0,09 im linearen Modell, vgl. Tab. 2 und 3) und somit eine Nichtbetrachtung des Zusammenhanges nach sich ziehen könnte.

Zuletzt ist festzuhalten, dass das AIC beim nichtlinearen Modell mit 12.235,52 deutlich vorteilhaft gegenüber dem AIC für das lineare Modell mit 13.598,34 abschneidet und somit mit der gesteigerten Modellkomplexität des nichtlinearen Modells eine maßgeblich bessere Modellgüte einhergeht.

## Diskussion

Der Wechsel von Schule zu Homeschooling im Zuge der Coronapandemie führte zu einer völlig neuen Situation für Eltern und Kinder, in der die Eltern einen großen Teil der Verantwortung für das Gelingen des Unterrichts im eigenen Zuhause übernahmen. Der Beitrag analysiert die elterlichen Erwartungen, dass innerhalb der nächsten sechs Monate Schulprobleme auftreten werden.

Entsprechend des skizzierten Forschungsstands sind Ressourcen des Lernens wie ein ausgeprägtes schulisches Selbstkonzept und eine hohe Sorgfältigkeit für die Bewältigung des Homeschoolings bzw. für etwaige Probleme, die sich aus Elternsicht daraus ergeben, wichtig. Dies passt zu der Art des Unterrichtens im Homeschooling, welches einem selbstständigen Arbeiten und damit der Form von Hausaufgaben sehr nahekommt. Schüler*innen mit einem ausgeprägten Selbstkonzept wird es leichter fallen, sich ohne oder mit nur wenig fremder Hilfe mit den Aufgaben zu befassen und diese zu bewältigen. Gerade für letzteres ist eine hohe Sorgfältigkeit ebenfalls hilfreich. Eine ausgeprägte Lesekompetenz führt zu weniger negativen Erwartungen seitens der Eltern, dass Schulprobleme auftreten können. Da selbstständiges Arbeiten mit viel Lesen verbunden ist, scheinen Schüler*innen, die gut lesen können, einen Vorteil zu haben. Ferner zeigt eine Betrachtung sowohl linearer als auch nichtlinearer Modellierung, dass die Natur des Zusammenhangs zwischen Lesekompetenz und den Erwartungen der Eltern keinem linearen Verlauf folgt und bei linearer Modellierung die Variable entsprechend nicht statistisch signifikant ist.

Für die Zusammenhänge zwischen der sozialen Herkunft und den Erwartungen der Eltern, dass Schulprobleme auftreten können, verweisen die berechneten Modelle hinsichtlich des ökonomischen Kapitals auf signifikante Zusammenhänge und zeichnen ein klares Bild: Eltern mit einem niedrigeren beruflichen Status erwarten ausgeprägtere Schulprobleme für ihre Kinder als Eltern mit einem höheren beruflichen Status. Eine mögliche Erklärung hierfür könnte sein, dass Eltern mit einem hohen beruflichen Status auf ihre eigene Fähigkeit vertrauen, ihren Kindern in schulischen Belangen helfen zu können. Entsprechend dem *Family Stress Model* könnten sich ökonomische Probleme, die sich durch die Pandemie für Berufe mit einem niedrigeren sozialen Status, wie beispielsweise Köch*in oder Frisör*in, ergeben, auf die Schüler*innen und damit auch auf die erwarteten Schulprobleme übertragen. Hingegen gibt es keinen signifikanten Zusammenhang zwischen der Nachbarschaftsstruktur und den erwarteten Schulproblemen aus Elternsicht. Dies ist nicht zuletzt wohl auf die gesetzlichen Vorgaben und die gesellschaftliche Praxis der Einschränkung sozialer Kontakte im Zuge des Lockdowns zurückzuführen.

Aufgrund der insgesamt geringen Ausprägung der RKI-Inzidenzwerte, die sicherlich auf den frühen Zeitpunkt der Durchführung der NEPS-C-Studie in der Pandemie zurückzuführen sind, sind deren Zusammenhänge für Schulprobleme in dieser Form nicht interpretierbar und sollten in nachfolgenden Studien nochmals genauer untersucht werden.

Im Rahmen dieses Beitrages werden nicht nur erste empirische Befunde präsentiert, welche Faktoren im Zusammenhang mit den elterlichen Erwartungen von Schulproblemen im mittelbaren Verlauf nach dem ersten Corona Lockdown stehen. Es wird auch aufgezeigt, dass explorative Analysen mit nichtlinearen Regressionstechniken Zusammenhänge aufdecken können, welche ggf. mit konventionellen linearen Regressionsmethoden unterkomplex dargestellt werden oder gar mangels hinreichender statistischer Signifikanz ganz zu verschwinden drohen.

Für zukünftige Studien zu diesem Thema wäre es interessant, die hier vorgestellten individuellen und Kontextmerkmale auf Zusammenhänge mit tatsächlichen Kompetenzen der Schüler*innen nach dieser ersten und nach den folgenden Schulschließungen im Zuge der Coronapandemie zu untersuchen. Dies wird mit den nächsten regulären Veröffentlichungen des NEPS möglich und wird zusätzliche Erkenntnisse zu den Folgen des Umgangs mit der Coronapandemie für Schüler*innen in Deutschland liefern.

### Supplementary Information





## Footnotes

1 Abweichungen bei den Kompetenzen ergeben sich durch die Datenselektion für das Matching mit den NEPS-C-Daten.

## References

[CR1] Akaike H (1983). Information measures and model selection. Bulletin of the International Statistical Institute.

[CR2] Anger, S., Bernhard, S., Dietrich, H., Lerche, A., Patzina, A., Sandner, M., & Toussaint, C. (2021). Schulschließungen wegen Corona: Regelmäßiger Kontakt zur Schule kann die schulischen Aktivitäten der Jugendlichen erhöhen. https://www.iab-forum.de/schulschliessungen-wegen-corona-regelmassiger-kontakt-zur-schule-kann-die-schulischen-aktivitaten-der-jugendlichen-erhohen/. Zugegriffen: 15. Sept. 2022.

[CR3] Bandura A (1973). Aggression. A social learning analysis.

[CR4] Bandura A, Walters RH (1963). Social learning and personality development.

[CR5] Beham-Rabanser M, Scaria-Braunstein K, Haring-Mosbacher SA, Forstner M, Bacher J, Aschauer W, Glatz C, Prandner D (2022). Arbeit und Familie im Covid-19-Alltag. Die Österreichische Gesellschaft während der Corona-Pandemie.

[CR15] Del Bello, C. L., Patacchini, E., & Zenou, Y. (2015). Neighborhood Effects in Education (IZA Discussion Paper No. 8956). https://papers.ssrn.com/sol3/papers.cfm?abstract_id=2589818. Zugegriffen: 26. Nov. 2021.

[CR6] Blossfeld H-P, Roßbach H-G (2019). *Education as a lifelong process: The German National Educational Panel Study (NEPS)* (Edition ZfE).

[CR7] Bol T (2020). Inequality in homeschooling during the Corona crisis in the Netherlands.

[CR8] Boudon R (1974). Education, opportunity, and social inequality: changing prospects in western society.

[CR9] Bourdieu P, Kreckel R (1983). Ökonomisches Kapital, kulturelles Kapital, soziales Kapital. Soziale Ungleichheiten.

[CR10] Bujard M, von der Driesch E, Ruckdeschel K, Laß I, Thönissen C, Schumann A, Schneider NF (2021). Belastungen von Kindern, Jugendlichen und Eltern in der Corona-Pandemie.

[CR11] Coleman JS (1988). Social capital in the creation of human capital. American Journal of Sociology.

[CR12] Conger RD, Ge X, Elder GH, Lorenz FO, Simons RL (1994). Economic stress, coercive family process, and developmental problems of adolescents. Child Development.

[CR13] Cordes, J. (2020). Umfrage während der Coronakrise. https://www.ots.at/presseaussendung/OTS_20200422_OTS0006/umfrage-waehrend-der-coronakrise. Zugegriffen: 26. Nov. 2021.

[CR14] Crane J (1991). The epidemic theory of ghettos and neighborhood effects on dropping out and teenage childbearing. American Journal of Sociology.

[CR16] Destatis (2021). Kreisfreie Städte und Landkreise nach Fläche, Bevölkerung und Bevölkerungsdichte am 31.12.2020. https://www.destatis.de/DE/Themen/Laender-Regionen/Regionales/Gemeindeverzeichnis/Administrativ/04-kreise.html. Zugegriffen: 26. Nov. 2021.

[CR17] Eickelmann, B., & Drossel, K. (2020). SCHULE AUF DISTANZ. Perspektiven und Empfehlungen für den neuen Schulalltag. Eine repräsentative Befragung von Lehrkräften in Deutschland. https://www.vodafone-stiftung.de/wp-content/uploads/2020/05/Vodafone-Stiftung-Deutschland_Studie_Schule_auf_Distanz.pdf. Zugegriffen: 26. Nov. 2021.

[CR18] Engzell P, Frey A, Verhagen MD (2021). Learning loss due to school closures during the COVID-19 pandemic. Proceedings of the National Academy of Sciences of the United States of America.

[CR19] Fahrmeir L, Kneib T, Lang S, Marx B (2007). Regression.

[CR20] forsa (2020). Das Deutsche Schulbarometer Spezial Corona-Krise.

[CR21] Ganzeboom, H. B. G. (2010). International Standard Classification of Occupations ISCO-08 With ISEI-08 scores. http://www.harryganzeboom.nl/isco08/isco08_with_isei.pdf. Zugegriffen: 26. Nov. 2021.

[CR22] Goldan J, Geist S, Lütje-Klose B, Fickermann D, Edelstein B (2020). Schüler*innen mit sonderpädagogischem Förderbedarf während der Corona-Pandemie. Herausforderungen und Möglichkeiten der Förderung – Das Beispiel der Laborschule Bielefeld. „Langsam vermisse ich die Schule …“. Schule während und nach der Corona-Pandemie.

[CR23] Grewenig E, Lergetporer P, Werner K, Wößmann L, Zierow L (2021). COVID-19 and educational inequality: how school closures affect low- and high-achieving students. European Economic Review.

[CR24] Grgic M, Bayer M (2015). Eltern und Geschwister als Bildungsressourcen? Der Beitrag von familialem Kapital für Bildungsaspirationen, Selbstkonzept und Schulerfolg von Kindern. Zeitschrift für Familienforschung.

[CR25] Hagenauer G, Oberwimmer K, Wallner-Paschon C, Itzlinger-Bruneforth U (2019). Zum Zusammenhang zwischen Hausaufgabenpraxis und Leseleistung: Ergebnisse aus PIRLS 2006, 2011 und 2016. Lesekompetenz der 10-Jährigen im Trend. Vertiefende Analysen zu PIRLS.

[CR26] Heintz-Martin VK, Langmeyer AN (2020). Economic situation, financial strain and child wellbeing in stepfamilies and single-parent families in Germany. Journal of Family and Economic Issues.

[CR27] Helbig M (2010). Neighborhood does matter! Soziostrukturelle Nachbarschaftscharakteristika und Bildungserfolg. Kölner Zeitschrift für Soziologie und Sozialpsychologie.

[CR28] Helbig, M., & Jähnen, S. (2018). Wie brüchig ist die soziale Architektur unserer Städte? Trends und Analysen der Segregation in 74 deutschen Städten (Discussion Paper P‑2018-001). https://bibliothek.wzb.eu/pdf/2018/p18-001.pdf. Zugegriffen: 26. Nov. 2021.

[CR29] Heller, S., & Zügel, O. (2020). „Schule zu Hause“ in Deutschland. Bestandsaufnahme im Corona-Lockdown aus Perspektive der Schüler/-innen und Eltern. https://www.telekom-stiftung.de/sites/default/files/files/media/publications/Ergebnisbericht-Homeschooling.pdf. Zugegriffen: 26. Nov. 2021.

[CR30] Helm C, Huber S, Loisinger T (2021). Was wissen wir über schulische Lehr-Lern-Prozesse im Distanzunterricht während der Corona-Pandemie? – Evidenz aus Deutschland, Österreich und der Schweiz. Zeitschrift für Erziehungswissenschaft.

[CR31] Helmke A (2012). Unterrichtsqualität und Lehrerprofessionalität.

[CR32] Hillmayr D, Täschner J, Brockmann L, Holzberger D (2021). Elternbeteiligung im schulischen Kontext. Potenzial zur Förderung des schulischen Erfolgs von Schülerinnen und Schülern.

[CR33] Huber SG, Günther PS, Schneider N, Helm C, Schwander M, Schneider J, Pruitt J (2020). COVID-19 und aktuelle Herausforderungen in Schule und Bildung.

[CR34] Hußmann A, Stubbe TC, Kasper D, Hußmann A, Wendt H, Bos W, Bremerich-Vos A, Kasper D, Lankes E-M, McElvany N, Stubbe TC, Valtin R (2017). Soziale Herkunft und Lesekompetenzen von Schülerinnen. IGLU 2016. Lesekompetenzen von Grundschulkindern in Deutschland im internationalen Vergleich.

[CR35] Jencks C, Mayer SE, Lynn LE, McGreary MGH (1990). The social consequences of growing up in a poor neighbor-hood. Inner-city poverty in the United States.

[CR36] Lin N, Dumin M (1986). Access to occupations through social ties. Social Networks.

[CR37] Lochner, B. (2020). Thüringer Familien in Zeiten von Corona – Wohlbefinden der Kinder, Herausforderungen des Homeschooling & Unterstützungsbedarfe der Eltern. Erste Befunde. https://www.forum-transfer.de/fileadmin/user_upload/20-04-25_Befr.Familien-1.Befunde.pdf. Zugegriffen: 26. Nov. 2021.

[CR38] Lockl K, Attig M, Nusser L, Wolter I (2021). Lernen im Lockdown: Welche Voraussetzungen helfen Schülerinnen und Schülern?.

[CR39] Ludewig U, Kleinkorres R, Schaufelberger R, Schlitter T, Lorenz R, König C, Frey A, McElvany N (2022). COVID-19 Pandemic and Student Reading Achievement – Findings from a&nbsp;School Panel Study. Front. Psychol.

[CR40] NEPS-Netzwerk (2020). Nationales Bildungspanel, Scientific Use File der Startkohorte Kindergarten.

[CR59] NEPS-Netzwerk (2021). NEPS Corona & Bildung. https://www.neps-data.de/Datenzentrum/Daten-und-Dokumentation/NEPS-C. Zugegriffen: 26. Nov. 2021.

[CR41] Nonnenmacher A (2009). Ist Arbeit eine Pflicht? Normative Einstellungen zur Erwerbsarbeit, Arbeitslosigkeit und der Einfluss des Wohngebiets.

[CR42] Nonnenmacher A, Oberwittler D, Rabold S, Baier D (2013). Zur Nachweisbarkeit von Kontexteffekten der sozialräumlichen Umgebung. Städtische Armutsquartiere – Kriminelle Lebenswelten?.

[CR43] Pohl, S., & Carstensen, C. H. (2012). *NEPS technical report—scaling the data of the competence tests* (NEPS Working Paper No., Bd. 14). Bamberg: Otto-Friedrich-Universität, Nationales Bildungspanel.

[CR44] Robert Koch-Institut (2021). Robert Koch-Institut COVID-19-Dashboard https://experience.arcgis.com/experience/478220a4c454480e823b17327b2bf1d4. Zugegriffen: 15. Mai 2021.

[CR45] Sari E, Bittmann F, Homuth C (2021). Explaining inequalities in homeschooling in Germany during the first COVID-19 Lockdown.

[CR46] Schneider T, Tully CJ (2006). Die Inanspruchnahme privat bezahlter Nachhilfe. Ein kaum beachtetes Thema in der Bildungsforschung. Lernen in flexibilisierten Welten. Wie sich das Lernen der Jugend verändert.

[CR47] Schönberger, K., & Koberg, T. (2016). Regionaldaten: Microm. https://www.neps-data.de/Portals/0/NEPS/Datenzentrum/Forschungsdaten/Regio_Microm.pdf. Zugegriffen: 26. Nov. 2021.

[CR48] Schult J, Mahler N, Fauth B, Lindner MA (2022). Did students learn less during the COVID-19 pandemic? Reading and mathematics competencies before and after the first pandemic wave. School Effectiveness and School Improvement.

[CR49] Steinmayr, R., & Christiansen, H. (2020). Erste Erkenntnisse zur Qualität von Homeschooling. https://www.tu-dortmund.de/nachrichtendetail/detail/erste-erkenntnisse-zur-qualitaet-von-homeschooling-3036/. Zugegriffen: 26. Nov. 2021.

[CR50] Stubbe TC, Kriegt M, Beese C, Jusufi D, Schwippert K, Kasper D, Köller O, McElvany N, Selter C, Steffensky M, Wendt H (2020). Soziale Disparitäten in den mathematischen und naturwissenschaftlichen Kompetenzen von Viertklässlerinnen und Viertklässlern. TIMSS 2019. Mathematische und naturwissenschaftliche Kompetenzen von Grundschulkindern in Deutschland im internationalen Vergleich.

[CR51] Täschner J, Holzberger D, Hillmayr D (2021). Elternbeteiligung als Potenzial zur Förderung des schulischen Erfolgs. DDS – Die Deutsche Schule.

[CR52] Weis M, Müller K, Mang J, Heine J, Mahler N, Reiss K, Reiss K, Weis M, Klieme E, Köller O (2019). Soziale Herkunft, Zuwanderungshintergrund und Lesekompetenz. PISA 2018. Grundbildung im internationalen Vergleich.

[CR53] Wildemann, A., & Hosenfeld, I. (2020). Bundesweite Elternbefragung zu Homeschooling während der Covid 19 Pandemie. Erkenntnisse zur Umsetzung des Homeschoolings in Deutschland. http://www.zepf.eu/wp-content/uploads/2020/06/Bericht_HOMEschooling2020.pdf. Zugegriffen: 26. Nov. 2021.

[CR55] Wood SN (2017). Generalized additive models: an introduction with R.

[CR56] Zinn S, Bayer M (2021). Subjektive Belastung der Eltern durch die Beschulung ihrer Kinder zu Hause zu Zeiten des Corona-bedingten Lockdowns im Frühjahr 2020. Zeitschrift für Erziehungswissenschaft.

[CR57] Züchner I, Jäkel HR (2021). Fernbeschulung während der COVID-19 bedingten Schulschließungen weiterführender Schulen: Analysen zum Gelingen aus Sicht von Schülerinnen und Schülern. Zeitschrift für Erziehungswissenschaft.

